# Facile synthesis of silver/silver thiocyanate (Ag@AgSCN) plasmonic nanostructures with enhanced photocatalytic performance

**DOI:** 10.3762/bjnano.8.277

**Published:** 2017-12-22

**Authors:** Xinfu Zhao, Dairong Chen, Abdul Qayum, Bo Chen, Xiuling Jiao

**Affiliations:** 1 School of Chemistry and Chemical Engineering, Shandong University, Jinan 250100, PR China; 2 National Engineering Research Center for Colloidal Materials, Shandong University, Jinan 250100, PR China

**Keywords:** Ag@AgSCN, degradation of oxytetracycline, plasmonic photocatalyst, stability

## Abstract

A nanostructured plasmonic photocatalyst, silver/silver thiocyanate (Ag@AgSCN), has been prepared by a simple precipitation method followed by UV-light-induced reduction. The ratio of Ag to silver thiocyanate (AgSCN) can be controlled by simply adjusting the photo-induced reduction time. The formation mechanism of the product was investigated based on the time-dependent experiments. Further experiments indicated that the prepared Ag@AgSCN nanostructures with an atomic ratio of Ag/AgSCN = 0.0463 exhibited high photocatalytic activity and long-term stability for the degradation of oxytetracycline (84%) under visible-light irradiation. In addition to the microstructure and high specific surface area, the enhanced photocatalytic activity was mainly caused by the surface plasmon resonance of Ag nanoparticles, and the high stability of AgSCN resulted in the long-term stability of the photocatalyst product.

## Introduction

In the past decade, water decontamination technology has attracted great attention due to the increasing health risk that water contamination poses to humankind. The removal of pollutants has been intensively investigated in recent years, and various methods such as adsorption, biodegradation, photocatalytic degradation and chemical oxidation have been developed [1–4]. Among them, photocatalytic degradation is considered as one of the most effective strategies due to its high removal efficiency and environmental friendlessness. For example, as a typical semiconductor, TiO_2_ exhibits high photocatalytic degradation performance against a large number of organic pollutants [5–7]. However, it is difficult to obtain a high photocatalytic activity under visible-light irradiation with TiO_2_ as a catalyst due to its wide bandgap of 3.2 eV, which limits its practical application. Therefore, the development of new photocatalysts with visible-light catalytic performance, high surface active sites and long life of separated electron and hole pairs, has become a hot research topic in recent years.

Ag-based semiconductors are well known due to their excellent visible-light catalytic properties, but their easy inactivation limits their utilization in practice. Aimed at this problem, a series of methods, such as doping, surface sensitization, heterojunctions and noble-metal plasma, have been adopted to improve the visible-light catalytic performance of Ag-based materials [8–10]. Among them, plasmonic photocatalysts consisting of Ag/AgX (X = Cl, Br, I) have exhibited improvement in separation of photogenerated electrons and holes [11–18]. However, these Ag-based photocatalysts, Ag/AgX (X = Cl, Br, I), suffer from instability during the recycling application, mainly due to the decomposition of AgX (X = Cl, Br, I) under irradiation [19–22]. Therefore, the development of visible-light catalysts with both high catalytic activity and long-term stability is of great importance.

As one of the Ag-containing semiconductor materials, AgSCN exhibits superior stability under irradiation [23–24]. The relatively large bandgap of AgSCN (3.4 eV) makes it only ultraviolet light active, largely limiting the wide utilization of the solar light energy spectrum. The addition of Ag on AgSCN structures can not only improve the utilization of visible light, but can also trap the photogenerated electrons to enhance the catalytic efficiency and stability. In the present work, a nanostructured Ag@AgSCN plasmonic photocatalyst was achieved by using hydrazine hydrate as a reducing agent, followed by a UV-light-induced reduction, in which the ratio of Ag to AgSCN can be controlled by simply adjusting the photo-induced reduction time. The degradation of a representative contaminate, oxytetracycline, which often exists in contaminated water, was used to test the photocatalytic performance of Ag@AgSCN. Compared with previous reports, the as-prepared product had regular morphology, more active sites and a higher specific surface area [23–28], which led to high photocatalytic activity. More importantly, the activity toward the degradation of oxytetracycline after the fifth cycle was almost the same as for the first cycle, which is notable for a Ag-based catalyst.

## Results and Discussion

### Ag@AgSCN nanostructures

**Figure 1:**
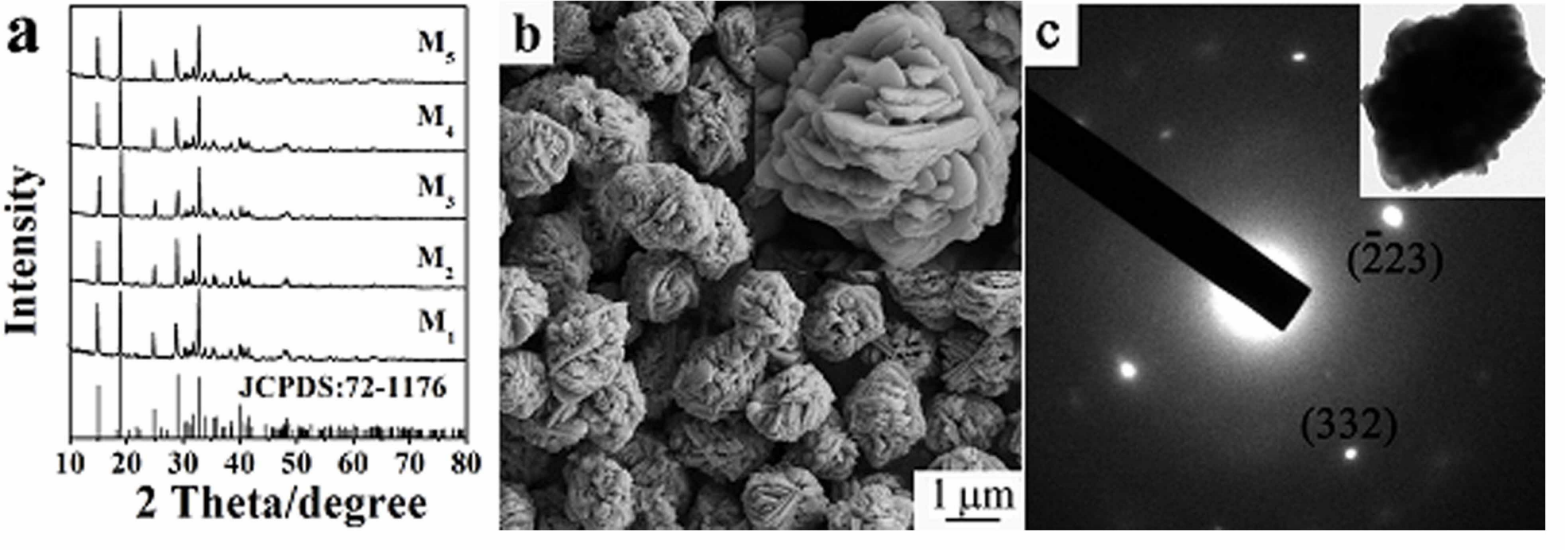
(a) XRD patterns of Ag@AgSCN nanostructures with different molar ratios of Ag to AgSCN. An (b) SEM image and (c) SAED pattern of the Ag@AgSCN nanostructure (sample M_2_).

Ag@AgSCN nanostructures were synthesized by a simple precipitation method, followed by UV-light-induced reduction. As shown in Figure 1a, all the XRD patterns of the Ag@AgSCN nanostructures formed at different irradiation times can be indexed to monoclinic AgSCN (JCPDS file, No. 72-1176). No obvious reflections of face-centered cubic Ag can be observed even with an irradiation time as long as 3 h, mainly due to the small amount of Ag derived from the high stability of AgSCN. An SEM image of the sample M_2_ is shown in Figure 1b as a representation. It can be seen that the product is sphere-like nanostructures with uniform size of ≈2.0 µm. Further observation of the high-resolution SEM image (inset of Figure 1b) reveals that the nanostructures are composed of close-packed nanoplates. The large nanoplates with a thickness of tens of nanometers firstly close arrange into crisscross structures, and there are many small plates in the crossed gap to form the sphere-like nanostructures. The corresponding EDS analysis indicates that only elemental Ag, C, N, and S are detected in the sample (Supporting Information File 1, Figure S1), showing the high chemical purity of the product. The selected area electron diffraction (SAED) pattern of a single particle (Figure 1c) displays a set of bright spots, obviously suggesting the high crystallinity of the product. Based on calculations, the spots can be indexed to diffraction from the 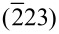 and (332) planes of the monoclinic AgSCN. The SAED pattern also reveals that the oriented attachment of the nanoplates composed the Ag@AgSCN nanostructures.

To determine the Ag content in the Ag@AgSCN nanostructures, elemental analysis was conducted, and the results are shown in Table 1. Before UV irradiation, the molar ratio of Ag to AgSCN in the product is 0.024. With the extension of the UV irradiation time, the content of Ag in the product gradually increases, and the molar ratio of Ag to AgSCN reaches 0.0775 after being irradiated for 3 h, indicating the slow reduction of AgSCN under UV irradiation.

**Table 1:** Elemental content of the synthesized Ag@AgSCN samples with different reduction times. In all calculation process, the molar value of S was representative of AgSCN.

Sample	Weight of Ag@AgSCN (mg)	Atomic content of S (%)	AgSCN (mol)^a^ × 10^−5^	Ag (mol)^b^ × 10^−7^	Molar ratio, Ag/AgSCN^c^

M_1_	2.233	18.987	1.325	3.177	0.024
M_2_	2.111	18.719	1.235	5.723	0.0463
M_3_	2.557	18.631	1.489	8.014	0.0538
M_4_	2.431	18.502	1.406	9.127	0.0649
M_5_	3.008	18.358	1.726	13.375	0.0775

^a^Molar content of AgSCN is calculated as the mole of AgSCN = *A*·*B*/32 (where *A* is the atomic % of S and *B* is the weight (mg) of Ag@AgSCN. ^b^Molar content of Ag is calculated according to Ag = (*B* – (*A*·*B*/32)·165.95)/107.87 (where *A* is the atomic % of S and *B* is the weight (mg) of Ag@AgSCN. ^c^Molar ratio of Ag/AgSCN is calculated as Ag/AgSCN = *B*/*A*.

**Figure 2:**
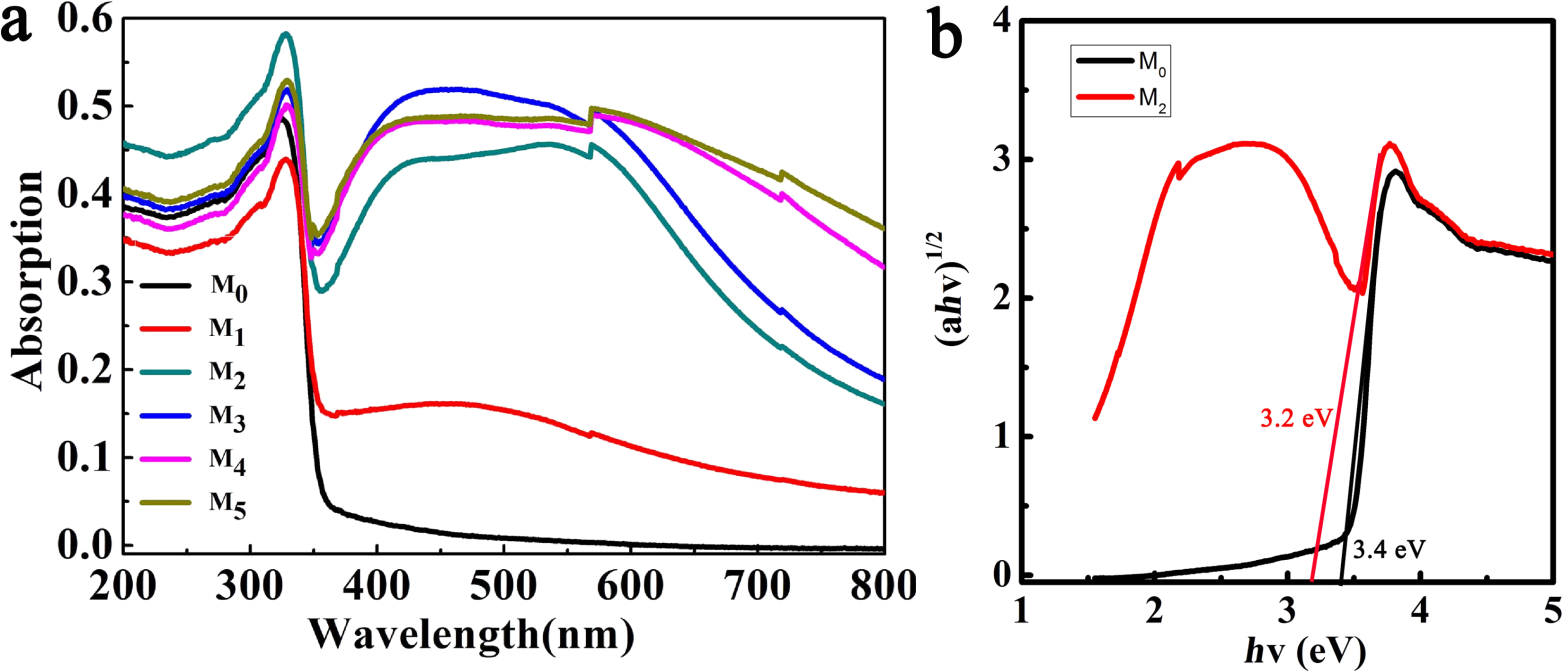
(a) UV–vis diffuse reflectance spectra of M_0_, M_1_, M_2_, M_3_, M_4_, M_5_. (b) Kubelka–Munk plots of M_0_ and M_2_.

UV–vis diffuse reflectance spectra of M_0_, M_1_, M_2_, M_3_, M_4_, and M_5_ are shown in Figure 2a. Here, the characteristic absorption of AgSCN appears at 200–350 nm and that from the surface plasmon resonance of Ag particles is above 350 nm. The absorption peak of silver nanoparticles becomes gradually stronger as the content of Ag increases, indicating that the sunlight utilization efficiency increases steadily. During UV irradiation, many defects are formed in AgSCN, including different interstitial sites and vacancies [29]. The presence of silver particles not only improves the photocatalytic efficiency, but also enhances the electric field strength around AgSCN due to the surface plasmon resonance, which in turn enhances the optical transition of midgap defect states of AgSCN. All these conditions contribute to the strong absorption of Ag@AgSCN in both the UV and visible region, which is beneficial for application as a visible-light catalyst. The bandgap of AgSCN can be determined according to the Kubelka–Munk equation, which is estimated as 3.4 eV for sample M_0_ (Figure 2b), and the valence band value of 1.12 eV is obtained from [30]. Therefore, the conduction band position can be determined to be −2.28 eV. The bandgap of sample M_2_ is estimated as 3.2 eV, where the change in the value of the bandgap of AgSCN may be due to the surface plasmon resonance of Ag nanoparticles under visible light. In this process, electrons in the metal from the Fermi level directly transfer to the conduction band of AgSCN, and the vacancies remain on the surface of the Ag particles. A dipole-based resonance energy can directly excite semiconductors to produce photogenerated electron–hole pairs that can improve the visible-light catalytic activity of AgSCN [31–32].

### Formation mechanism of Ag@AgSCN nanostructures

**Figure 3:**
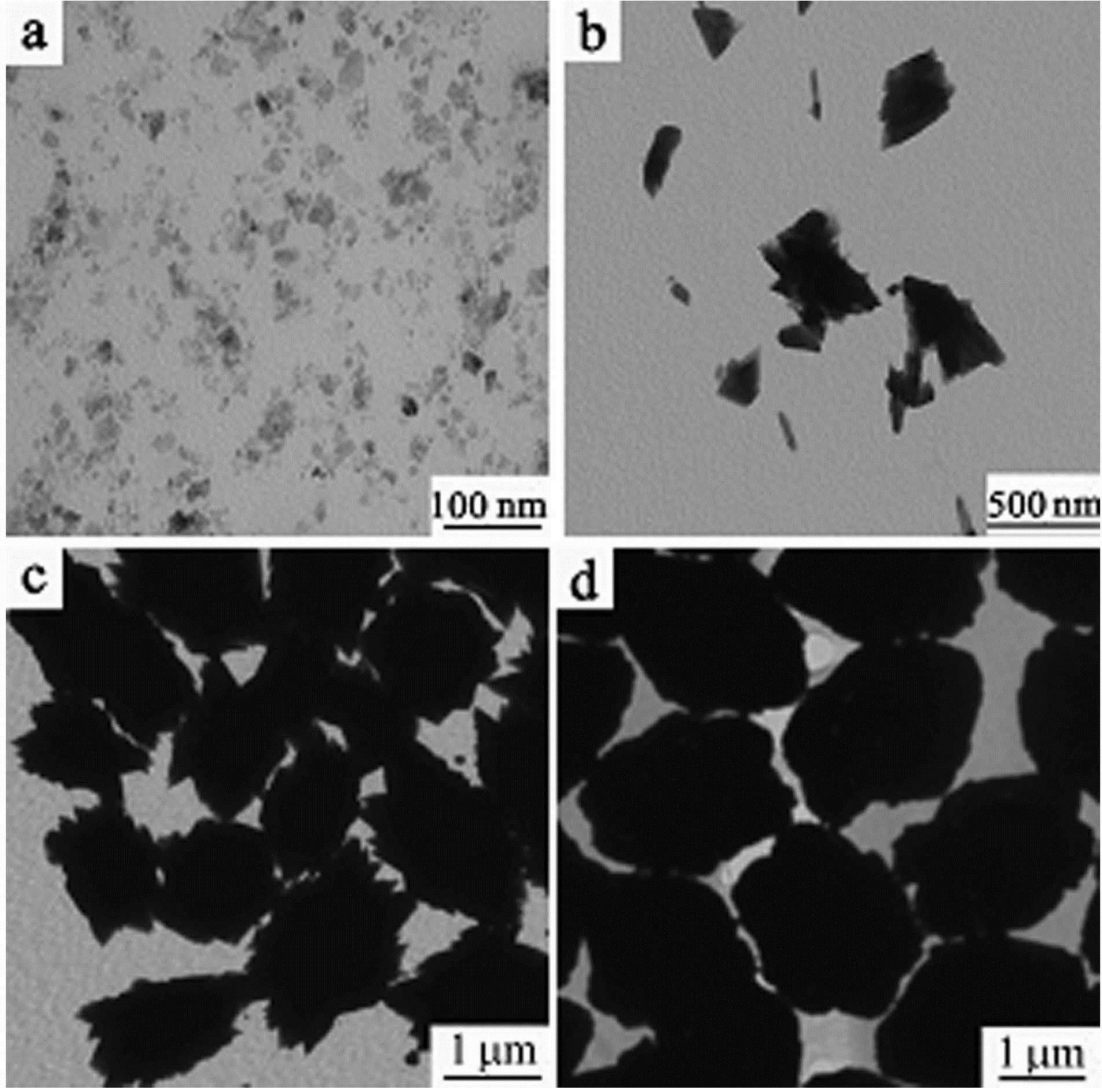
TEM images of precipitated samples formed after addition of the AgNO_3_ solution after (a) 2 min, (b) 6 min, (c) 10 min, and (d) 20 min.

**Figure 4:**
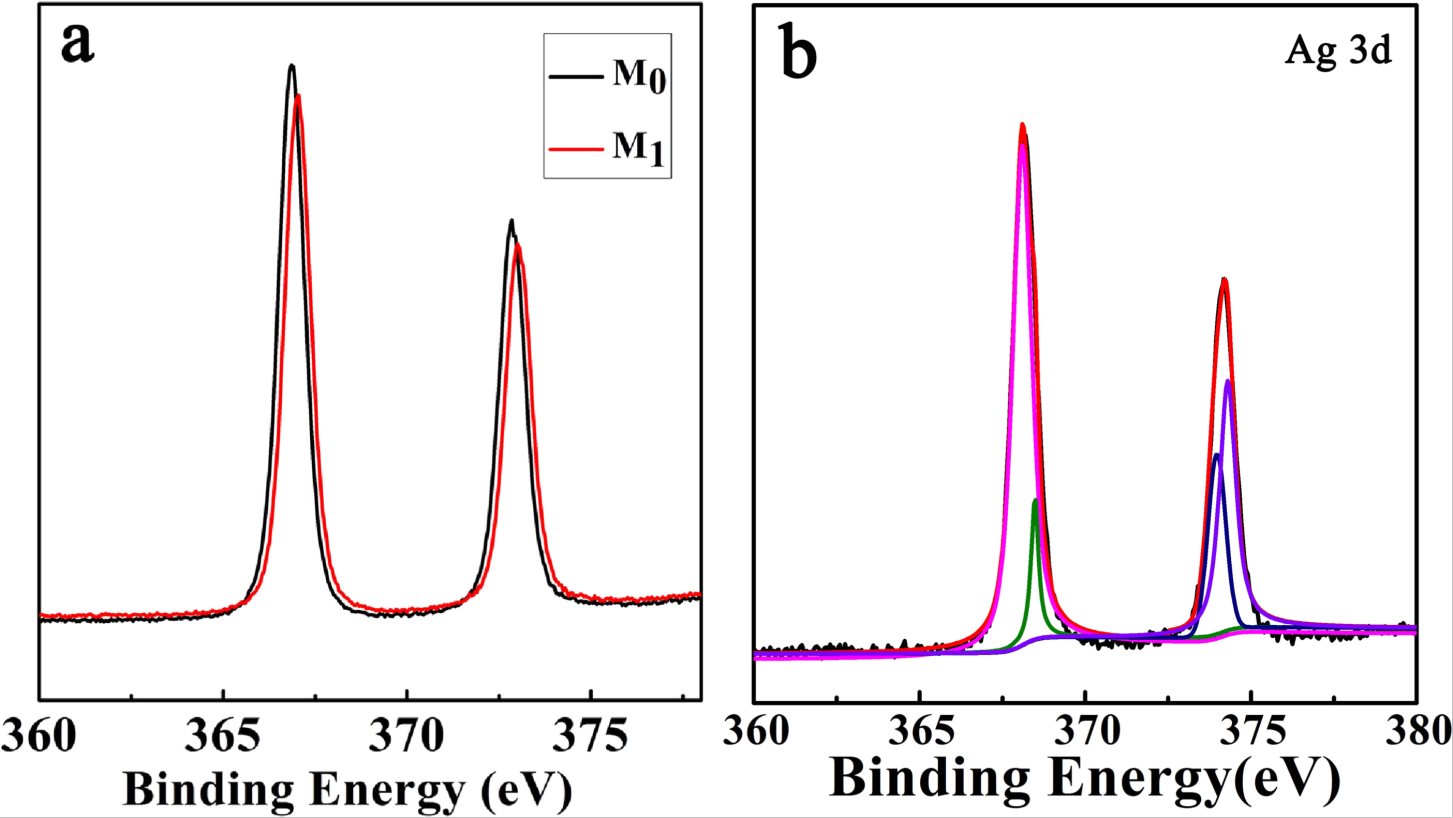
(a) XPS spectra of M_0_ and M_1_; (b) XPS peaks of Ag 3d_5/2_ and Ag 3d_3/2_ of M_1_.

To investigate the formation mechanism of Ag@AgSCN nanostructures, a time-dependent precipitation experiment was conducted. It can be seen that small AgSCN nanoplates with size of ≈10 nm were formed in 2 min due to the low solubility product constant (*K*_sp_) of AgSCN (Figure 3a). These nanoplates rapidly aggregated due to the high surface energy, and the crystal growth occurred simultaneously (Figure 3b). At this time, the dropwise addition of the AgNO_3_ solution finished. The oriented-aggregates with a size of ≈1.5 µm were formed in 10 min (Figure 3c). Further extending the reaction time led to the gradual increase in the size of the nanostructures, and the sphere-like nanostructures with size of ≈2 µm were finally obtained (Figure 3d). Certainly, because of the existence of N_2_H_4_·H_2_O, a small amount of Ag was formed at the same time, but it cannot be distinguished from the TEM image. So the XPS spectra of the samples obtained with and without N_2_H_4_·H_2_O were compared (Figure 4a). As to the AgSCN nanostructures formed without N_2_H_4_·H_2_O, the bonding energy of Ag 3d_3/2_ and Ag 3d_5/2_ located at 373.35 eV and 367.8 eV with a symmetrical profile, indicated that there was no Ag^0^ in the sample. For M_1_ (Figure 4b), the peaks of Ag 3d_5/2_ and Ag 3d_3/2_ can be divided into two different peaks at 368.2, 368.0 eV and 374.3, 373.9 eV, respectively. The peaks at 368.2 and 374.3 eV can be attributed to Ag^0^, indicating the presence of Ag^0^ [33]. The elemental analysis results shown in Table 1 also indicate the existence of a small amount of Ag^0^ in the precipitated products.

Further experiments indicated that N_2_H_4_·H_2_O did not affect the morphology of the product. As shown in Figure S2a (see Supporting Information File 1), the phase-pure AgSCN with the same morphology as the precipitated product was formed without the addition of N_2_H_4_·H_2_O while other conditions were held constant, indicating the existence of N_2_H_4_·H_2_O and the formation of the small amount of Ag had no effect on the morphology of the product. Figure S2b (see Supporting Information File 1) shows the SEM image of the sample obtained without polyvinylpyrrolidone (PVP). The product was composed of nanoparticles with of tens of nanometers in diameter with a large size distribution. This result revealed that the formation of the nanoplate units which composed the Ag@AgSCN nanostructures was directed by PVP. According to the previous studies, it was proposed that PVP can selectively adsorb on certain crystal planes and then lead to the formation of AgSCN nanoplates [34]. The rapid addition of the AgNO_3_ solution (instead of dropwise addition) resulted in the formation of crisscross nanostructures composed of nanoplates without small plates in the cross gap (see Supporting Information File 1, Figure S2c). BET measurements were used to illustrate the specific surface area of the nanostructured Ag@AgSCN, which was 4.04 m^2^·g^−1^, while the BET specific surface area of a sample without PVP (see Supporting Information File 1, Figure S2b) was only 2.88 m^2^·g^−1^.

Based on the above results, the formation process of Ag@AgSCN nanostructures can be summarized. The AgSCN nanoplates were quickly formed under the direction of PVP once the AgNO_3_ solution was added into the solution (Figure 3a). Then rapid aggregation of the nanoplates occurred to reduce the total surface energy, with the simultaneously growth of the AgSCN plates (Figure 3b). The nanoplates were arranged in a face-to-face stacking manner, and the tetragonal star-shaped Ag@AgSCN nanostructures composed of nanoplates were formed. After that, the addition of the AgNO_3_ solution finished and the homogeneous nucleation stopped due to the decrease in concentration of the Ag(I) ions. Then, the heterogeneous nucleation on the nanoplate surface occurred and some small nanoplates gradually filled in the crisscross gap of the star-shaped Ag@AgSCN nanostructures. At last, the sphere-like Ag@AgSCN nanostructures composed of nanoplates were formed.

In the following UV-light-induced reduction process, the content of Ag gradually increased with increased irradiation time. Due to the high stability, it is difficult for phase-pure AgSCN to be reduced to Ag by UV-light irradiation, but the existence of a small amount of Ag in the above precipitate can promote the transformation from AgSCN to Ag. Finally, the Ag@AgSCN nanostructures with different Ag content were obtained by adjusting the irradiation time of the UV light.

### Visible-light catalytic performance

**Figure 5:**
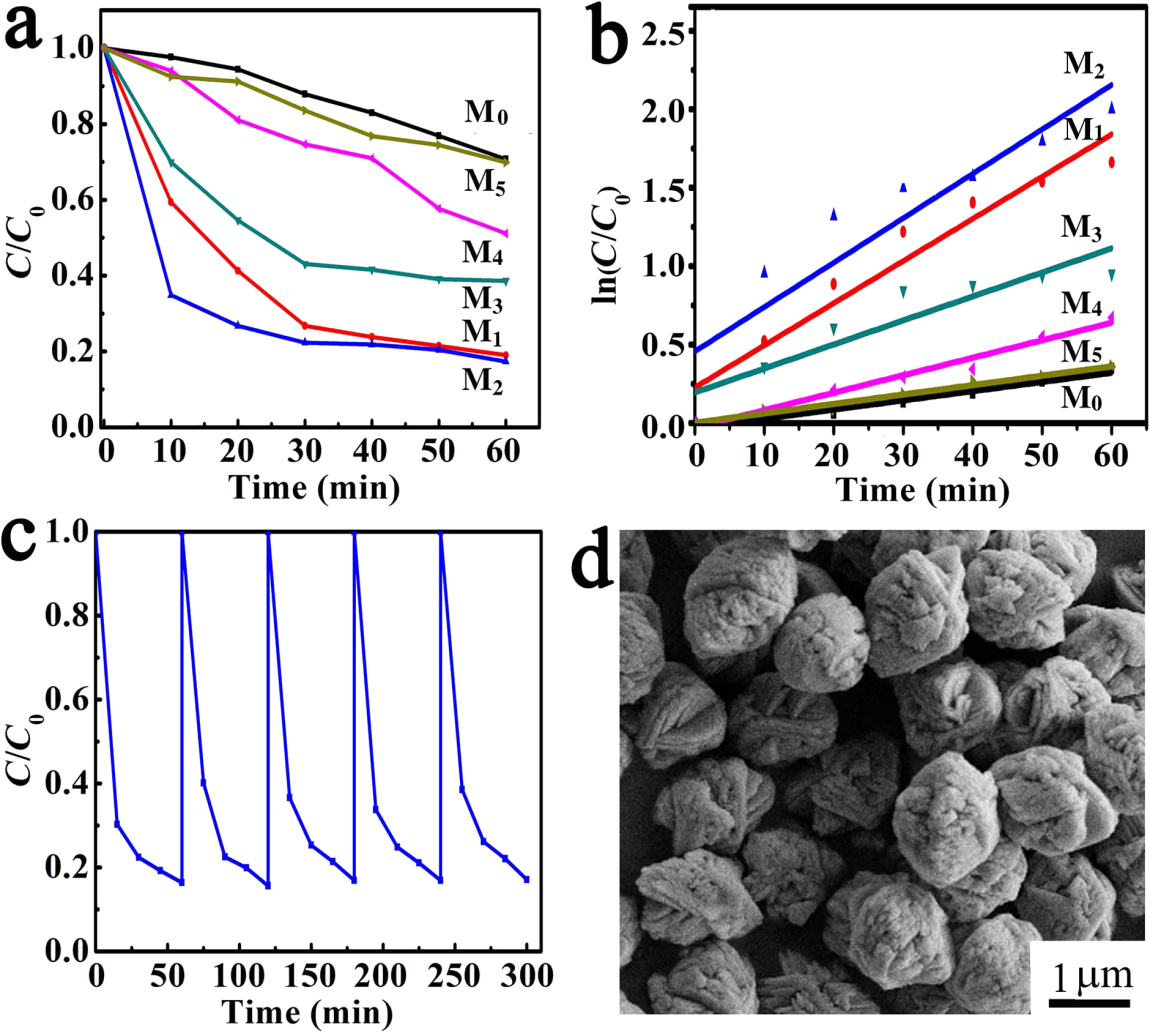
(a) Photocatalytic degradation of oxytetracycline over M_0_, M_1_, M_2_, M_3_, M_4_, M_5_. (b) Kinetic curves for the oxytetracycline degradation with M_0_, M_1_, M_2_, M_3_, M_4_, M_5_. (c) The cycle curves of oxytetracycline degradation using M_2_ as a photocatalyst. (d) TEM images of M_2_ after five cyclic experiments.

The visible-light catalytic activity of the samples was evaluated by measuring the degradation rate of oxytetracycline (20 mg·L^−1^) under a halogen lamp (400 W) using phase-pure AgSCN as a reference. The oxytetracycline concentration variation with irradiation time for the M_0_, M_1_, M_2_, M_3_, M_4_ and M_5_ catalysts are shown in Figure 5a. The degradation rates were 29%, 81%, 84%, 61%, 49%, and 30%, respectively, in 60 min according to the absorption intensity at 364 nm. When M_2_ was used as a photocatalyst, the maximum degradation rate was obtained. The time-dependent UV–vis spectrum of the oxytetracycline solution over M_2_ is shown in Supporting Information File 1, Figure S3. Furthermore, the degradation efficiency of Ag@AgSCN was much higher than that of the pure AgSCN, mainly due to the localized surface plasmon resonance state of Ag particles in the visible-light region. The photogenerated electrons of AgSCN will be captured by Ag particles, rather than recombination with holes. The dipole character of Ag particles in the plasma can promote the separation of electrons and holes in AgSCN under visible light. The electrons will quickly transfer onto the surface of Ag nanoparticles, while the holes will congregate on the surface of AgSCN [35]. On one hand, with increasing Ag content in the product, the recombination of electrons and holes is inhibited, and the catalytic efficiency would be enhanced. But on the other hand, the formation of excess Ag on the surface of AgSCN particles would partially cover the active sites and reduce the adsorption of oxytetracycline on the AgSCN particle surface. Furthermore, the presence of excess Ag particles will decrease the absorption of light and reduce the production of electrons and holes. All of these phenomena led to the decrease of the degradation efficiency [36–37]. Therefore, with the increase of the Ag content in the Ag@AgSCN nanostructures, the degradation efficiency firstly increased and then decreased. The Langmuir–Hinshelwood model was used to investigate the kinetic behaviors of the photocatalyst for degradation of oxytetracycline [38]. Figure 5b showed that the photodegradation process of oxytetracycline nearly complied with the first-order kinetics, and the corresponding reaction rate constants (*K*_a_) with M_0_, M_1_, M_2_, M_3_, M_4_ and M_5_ as catalysts were 0.0058, 0.0269, 0.02832, 0.01531, 0.01115, 0.006 min^−1^, respectively. The value of *K*_a_ for M_2_ was 4.8-fold faster than that of the bare AgSCN, so the existence of an appropriate amount of Ag played a prominent role in the photodegradation process. Electrochemical impedance spectrum (EIS) was used to illustrate the rate of charge transfer for the photocatalysts. Nyquist plots of M_0_, M_1_, M_2_, M_3_, M_4_, M_5_ were shown in Figure S4 (see Supporting Information File 1), the circular radius of plot over M_2_ in the high-frequency region was much smaller than that of other catalysts, which suggested the smallest charge transfer resistance for M_2_, while the charge transfer resistance for M_0_ was the largest. That can be attributed to the presence of Ag particles and the promotion of the separation of photogenerated carriers.

The stability of the plasmonic photocatalyst M_2_ was investigated by the degradation of oxytetracycline, which was of practical importance for application. As shown in Figure 5c, about 84% oxytetracycline was degraded in 60 min in the fifth cycle, and the efficiency was almost the same as for the first cycle. Further investigations revealed that after five cyclic experiments, the microstructure of Ag@AgSCN had no obvious changes except a somewhat smoother Ag@AgSCN nanostructure surface (Figure 5d). The composition of Ag, S, C, N in M_2_ hardly changed in the EDS analysis (see Supporting Information File 1, Figure S5), indicating that AgSCN cannot be reduced in this period of time under visible-light irradiation. Elemental analysis of the Ag@AgSCN after cyclic experiments were also performed. The result showed that the molar ratio of Ag to AgSCN is 0.047, which was near the value in the fresh M_2_. All these results indicated that the prepared Ag@AgSCN nanostructures showed long-term stability as a photocatalyst. For Ag@AgSCN, Ag particles coated the surface of AgSCN uniformly, which trapped the photogenerated electrons and protected AgSCN from being reduced. Compared with the previously reported silver halogen photocatalysts (Ag/AgX (X = Cl, Br, I)), the present Ag@AgSCN nanostructures exhibited superior catalytic stability [13–14]. This was mainly due to the higher stability of AgSCN than the AgX (X = Cl, Br, I). Also, the stronger electron affinity of SCN^0^ as compared to Br^0^ and Cl^0^, and the more easy combination of SCN^−^ with holes as compared to Cl^−^ and Br^−^ can improve the photocatalytic stability of Ag@AgSCN nanostructures.

**Figure 6:**
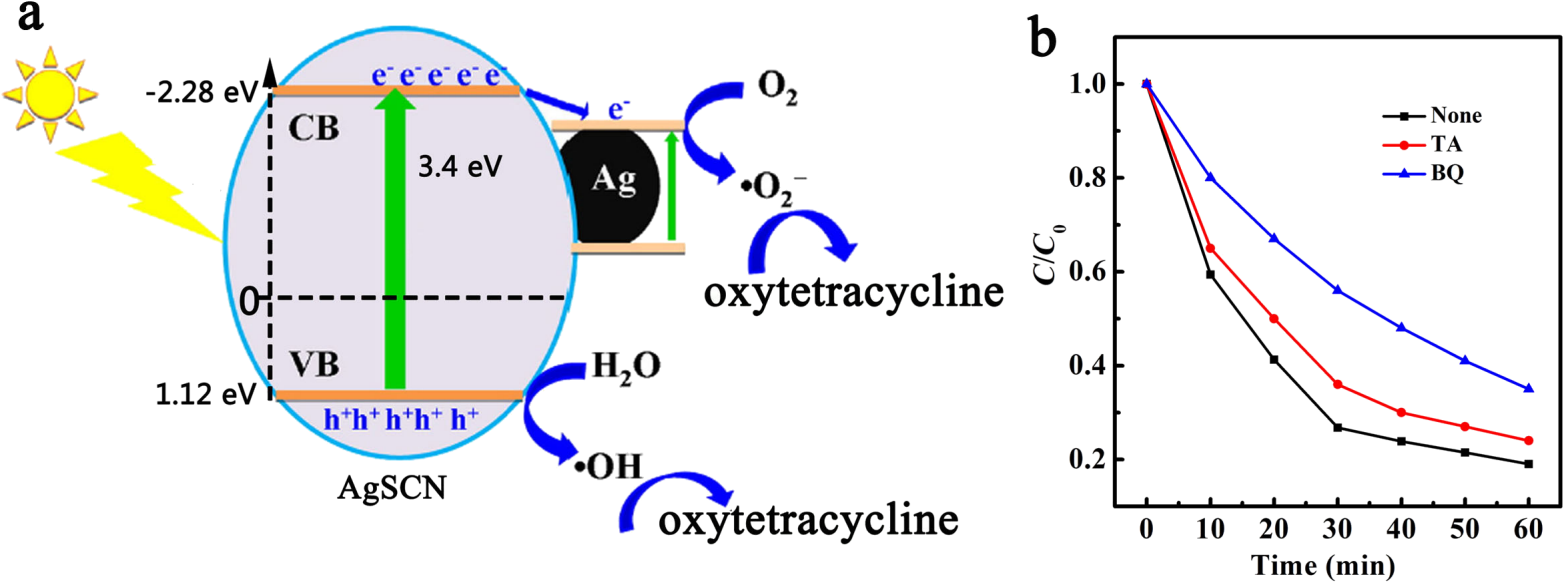
(a) Photocatalysis mechanism of the Ag@AgSCN plasmonic photocatalyst. (b) Photocatalytic degradation of oxytetracycline in the absence and presence of scavengers.

Based on the above discussion, Ag nanoparticles located on AgSCN played a decisive role in the photocatalytic efficiency and stability of Ag@AgSCN. For one, Ag nanoparticles can absorb the visible light, which enhances the utilization of visible light compared with pure AgSCN. On the other hand, the presence of Ag particles effectively reduced the recombination of photogenerated electrons and holes, improving the efficiency and stability of the photocatalyst. The surface plasmon resonance of Ag nanoparticles occurred under visible light, and electrons in the metal from the Fermi level directly transferred to the conduction band of AgSCN, whereas the vacancies were left on the surface of the Ag particles. The dipole-based resonance energy can directly excite semiconductors to produce photogenerated electron–hole pairs. The presence of Ag nanoparticles can not only improve the photocatalytic efficiency, but also can trap photogenerated electrons, slow the reduction of AgSCN, and enhance the stability of Ag@AgSCN. The degradation mechanism suggested that electrons of Ag@AgSCN were prompted to the conduction band and holes were left in the valence band of AgSCN under irradiation (Figure 6a). The electrons were rapidly trapped by Ag nanoparticles, then captured by dissolved O_2_, resulting in the formation of •O_2_^−^ radicals, which can decompose oxytetracycline. The photogenerated holes can oxidize H_2_O into •OH, which could also oxidize oxytetracycline. To investigate the effect of •O_2_^−^ radicals, 1, 4-benzoquinone (BQ), which was an efficient scavenger for •O_2_^−^ [39], was added to the solution. The degradation efficiency of oxytetracycline was significantly suppressed as shown in Figure 6b. Additionally, the presence of •OH was confirmed by addition of terephthalic acid (TA), which was a •OH scavenger [40]. The degradation efficiency of oxytetracycline was also slowed, but was higher than that of the rate with BQ. This revealed that the •O_2_^−^ and •OH both contributed to the decomposition of oxytetracycline, but •O_2_^−^ was the main active species in the photocatalytic process.

## Conclusion

Ag@AgSCN nanostructures composed of nanoplates were synthesized through a simple precipitation method, followed by UV-light irradiation. The sphere-like Ag@AgSCN nanostructures went through the formation of AgSCN nanoplates and their oriented aggregation, as well as simultaneous crystal growth. PVP played a critical role in the formation of 2D AgSCN units. The content of Ag in Ag@AgSCN nanostructures can be controlled by simply adjusting the irradiation time of the UV light. The catalytic performance of the nanostructure was evaluated by degradation of oxytetracycline under visible-light irradiation. The Ag@AgSCN nanostructures showed high photocatalytic activity due to their surface plasmon resonance effects. When the molar ratio of Ag/AgSCN was 0.0463, the catalytic rate reached to the maximum and the value of *K*_a_ was 4.8-fold faster than that of the bare AgSCN. Most importantly, the prepared Ag@AgSCN nanostructures also exhibited superior photocatalytic stability compared to the previously reported silver halogen plasma catalyst, which make it more suitable for practical application.

## Experimental

### Chemicals

Silver nitrate (AgNO_3_, Shanghai Chemical Co.) and ammonium thiocyanate (NH_4_SCN, Tianjin Reagent Co.) were used as precursors for the synthesis of silver/silver thiocyanate nanostructures (Ag@AgSCN). Polyvinylpyrrolidone (PVP, K30, Beijing Reagent Co.) and hydrazine hydrate (N_2_H_4_·H_2_O, 85 wt %, Shanghai Chemical Co.) were used as stabilizer and reducing agent, respectively. For the photocatalytic test, oxytetracycline was purchased from Sinopharm Chemical Co. Ltd (China). All chemicals were used as received without further purification.

### Synthesis of Ag@AgSCN nanostructures

Ag@AgSCN nanostructures were prepared by photoreducing AgSCN nanoparticles. Due to the slow reducing rate of AgSCN under UV–vis light irradiation, the AgSCN nanoparticles (with a small amount of Ag nuclei) were firstly synthesized as follows. At first, 50 mL of deionized water, 5.55 g of PVP, 0.38 g of NH_4_SCN and 2 mL N_2_H_4_·H_2_O (85 wt %) were sequentially added to the beaker, and then 10 mL solution of AgNO_3_ (0.5 M) was injected drop by drop via a pipette. The addition of the AgNO_3_ solution lasted for ≈6 min, after that the beaker was kept at 30 °C for 30 min under stirring and AgSCN nanostructures with a small amount of Ag nuclei were obtained (labeled as M_1_). The precipitate was collected by centrifugation and washed with water several times and then dried at 60 °C for 6 h in a vacuum drying box.

Next, 0.1 g of the above-described nanostructures was dispersed in 50 mL deionized water by ultrasonication. To establish the adsorption–desorption equilibrium, the dispersion was stirred for 30 min under dark prior to the light irradiation. Then the production was performed under UV-light irradiation by using a halogen lamp (400 W). The reaction lasted for 1, 2, 2.5, and 3 h to adjust the ratio of Ag to AgSCN (which are respectively labeled as M_2_, M_3_, M_4_, M_5_). The product was collected by centrifugation at 4000 rpm for 5 min and washed with water for three times. In this process, part of AgSCN was transferred to Ag by photoreduction with the color of the solid changing from milk white to pale red, red brown and black. The samples were dried at 60 °C for 6 h in a vacuum drying box. Then the Ag@AgSCN nanostructures with different molar ratio of Ag:AgSCN were obtained.

As a comparison, the pure AgSCN nanomaterial (labeled as M_0_) was also prepared using the same procedure as preparing AgSCN with a small amount of Ag except the addition of N_2_H_4_·H_2_O.

### Characterization

The morphology and microstructure of the samples were characterized using a field-emission scanning electron microscope (FE-SEM, JSM-6700F), a transmission electron microscope (TEM, JEM 100-CXII) with an accelerating voltage of 80 kV, and a high-resolution TEM (HRTEM, GEOL-2010) with an accelerating voltage of 200 kV. Also, powder X-ray diffraction (XRD) patterns were collected on an X-ray diffractometer (Rigaku D/Max 2200 PC) with a graphite monochromator and Cu Kα radiation (λ = 0.15148 nm), while the tube voltage and electric current were held at 40 kV and 20 mA. The composition of the samples was tested by Vario EL III organic elemental analyzer (Germany Elmentar Company). Ultraviolet–visible (UV–vis) diffuse reflectance spectra (DRS) were recorded on an Agilent Cary 100 UV–vis spectrophotometer coupled to an integrating sphere with BaSO_4_ as reference. X-ray photoelectron spectra (XPS) were recorded on a Perkin-Elmer PHI-5300 ESCA spectrometer with a pass energy of 35.75 eV and an Al Kα line excitation source. N_2_ adsorption–desorption isotherms were determined by using a Quadrasorb SI apparatus to obtain the Brunauer–Emmett–Teller (BET) surface area. Electrochemical impedance spectroscopy (EIS) was recorded on a CHI660A electrochemical workstation (CH Instrument Company, Shanghai, China).

### Photocatalytic performance test

The photocatalytic performance of the Ag@AgSCN nanostructures was evaluated by degradation of oxytetracycline (20 mg/L aqueous solution) at ambient temperature and pressure. To establish the adsorption–desorption equilibrium, the dispersion was stirred for 30 min under dark prior to the light irradiation. The degradation was performed under visible-light irradiation by using a halogen lamp (400 W) with a UV cutoff filter (λ ≥ 420 nm). The halogen lamp spectrum is commonly used to simulate the sunlight spectrum, in which the visible light is about 60–80%. An ≈3 mL aliquot was taken every 10 min and centrifuged to remove the dispersed photocatalyst, and the supernatant was transferred to a quartz cuvette to measure the UV–vis absorption spectrum. The concentration change of oxytetracycline was monitored according to its maximum absorbance at 364 nm (λ_max_) recorded by a Perkin-Elmer Lambda-35 UV–vis spectrometer.

## Supporting Information

File 1 Additional experimental data.
The Supporting Information contains details of the AgSCN characterization, Nyquist plots, EDS spectrum of different Ag/AgSCN samples, and UV–vis absorption spectra of oxytetracycline solutions with M_2_ as a catalyst.
