# Supplementary material for: Facile synthesis of silver/silver thiocyanate (Ag@AgSCN) plasmonic nanostructures with enhanced photocatalytic performance
Source: Beilstein J Nanotechnol. 2017 Dec 22;8:2781–9. doi: 10.3762/bjnano.8.277 (PMC5753043; doi:10.3762/bjnano.8.277)
Supplement: File 1 — Additional experimental data. The Supporting Information contains details of the AgSCN characterization, Nyquist plots, EDS spectrum of different Ag/AgSCN samples, and UV–vis absorption spectra of oxytetracycline solutions with M2 as a catalyst. [file Beilstein_J_Nanotechnol-08-2781-s001.pdf]

Supporting Information
for

Facile synthesis of silver/silver thiocyanate (Ag@AgSCN) plasmonic nanostructures with enhanced photocatalytic performance

Xinfu Zhao[1], Dairong Chen[1,2], Abdul Qayum[1], Bo Chen*[2] and Xiuling Jiao*[1,2]

Address: [1]School of Chemistry and Chemical Engineering, Shandong University, Jinan 250100, PR China and [2]National Engineering Research Center for Colloidal Materials, Shandong University, Jinan 250100, PR China

Email: Bo Chen - sduchenbo@126.com, Xiuling Jiao - jiaoxl@sdu.edu.cn

* Corresponding author

Additional experimental data

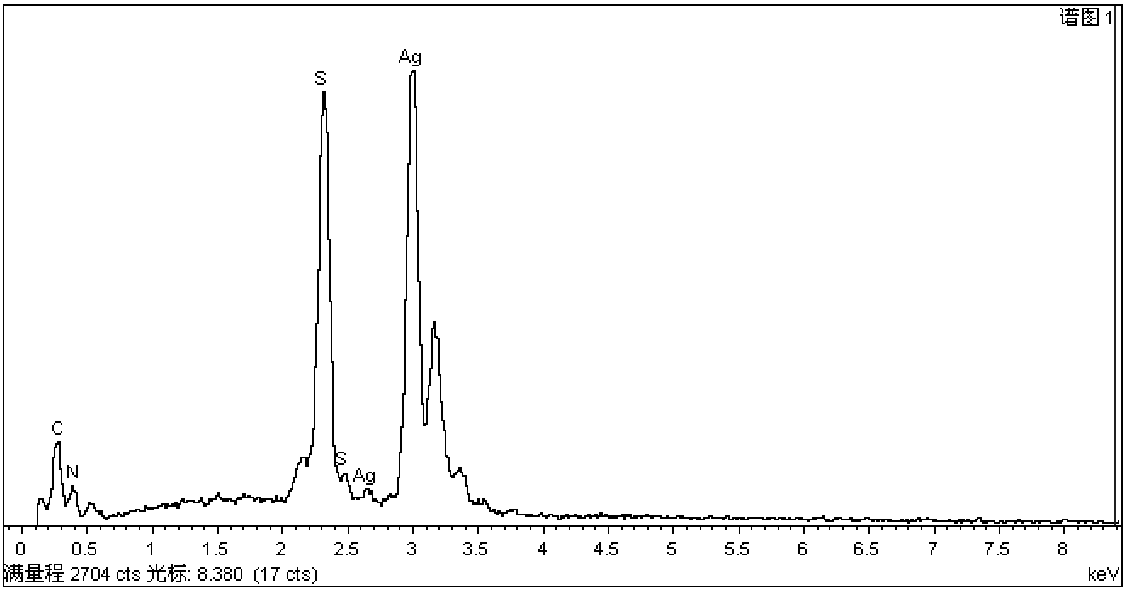

Figure S1: EDS spectrum of AgSCN nanoparticles.

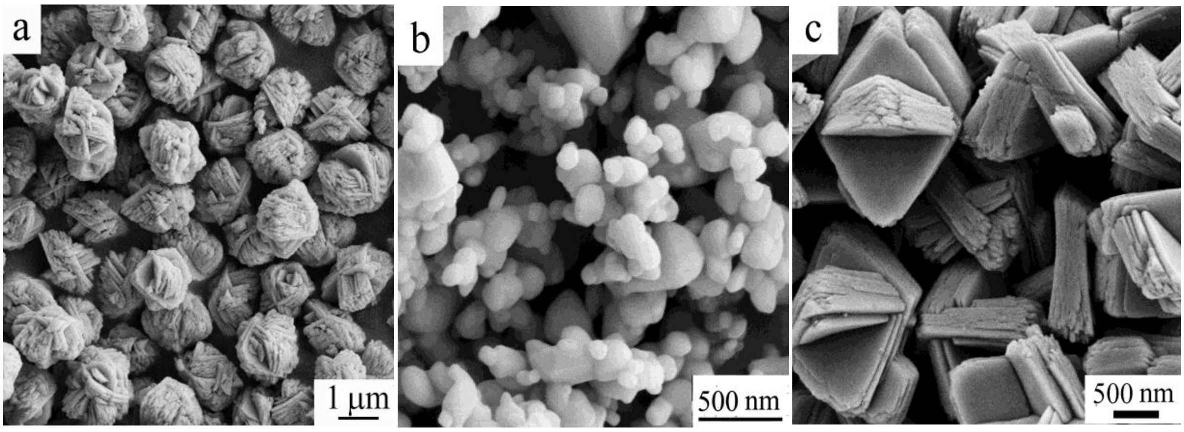

Figure S2: SEM images of AgSCN particles produced with the presence of PVP (a), and without the addition of PVP (b), with rapid addition of $AgNO_3$ solution instead of dropwise addition (c).

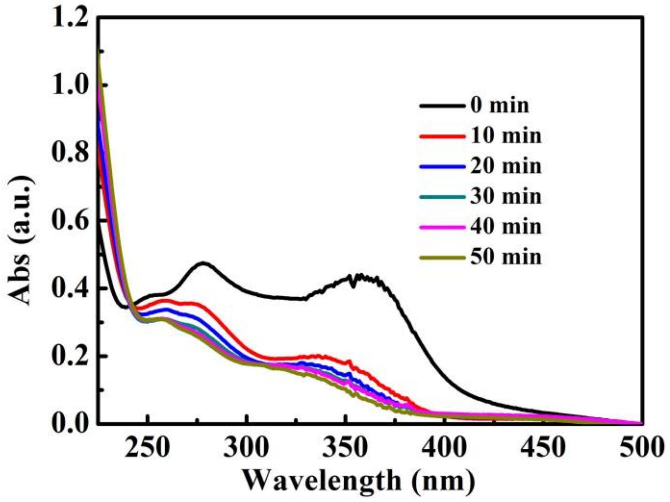

Figure S3: Time dependent variation of UV–vis absorption spectrum of oxytetracycline solution with M_2 as catalyst.

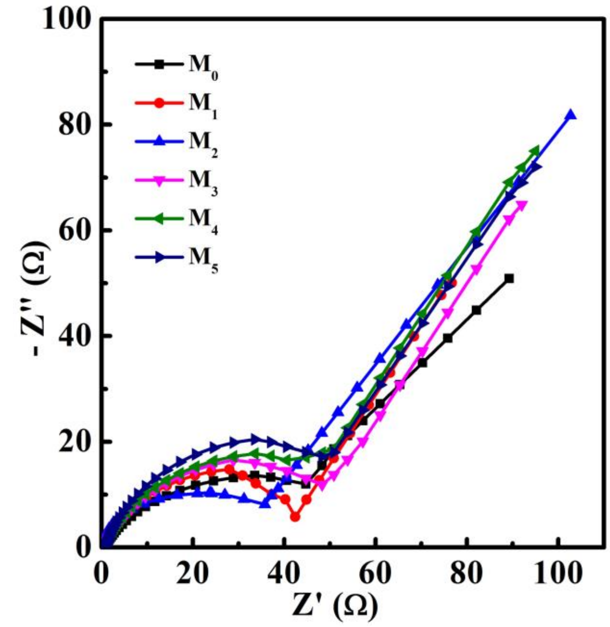

Figure S4: Nyquist plots of M_0, M_1, M_2, M_3, M_4, M_5 in 0.1 M NaOH solution with the frequency range of 100 kHz – 0.01 Hz.

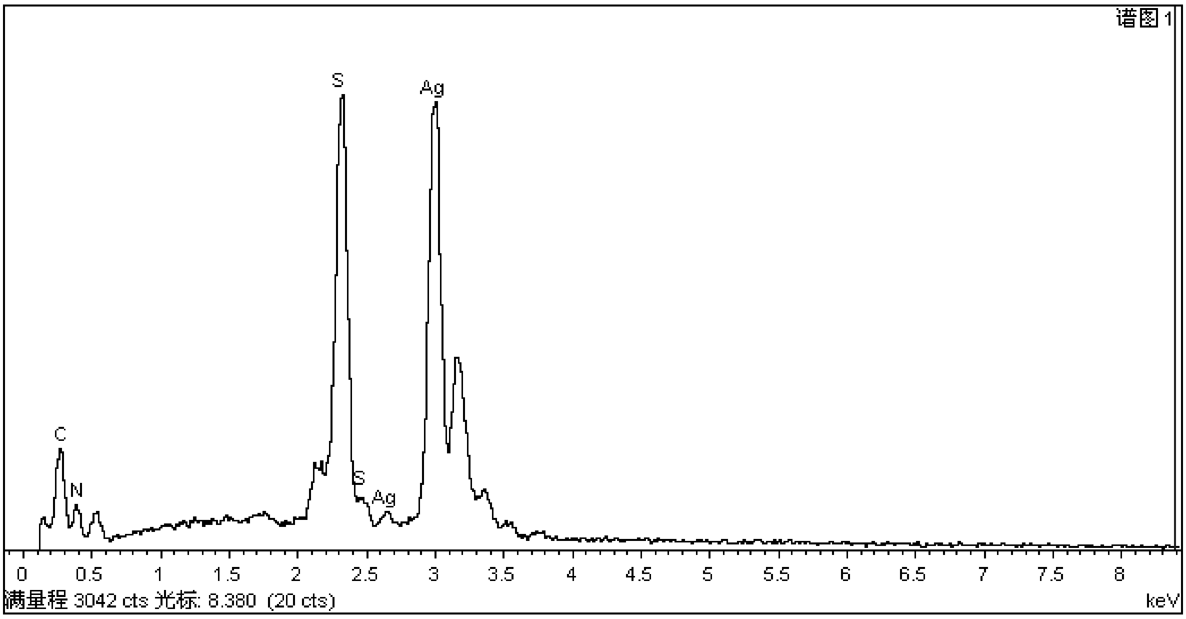

Figure S5: EDS spectrum of M_4 after five cyclic experiments.
